# Granulocyte-colony stimulating factor ameliorates di-ethylhexyl phthalate-induced cardiac muscle injury via stem cells recruitment, Desmin protein regulation, antifibrotic and antiapoptotic mechanisms

**DOI:** 10.1007/s10735-023-10137-6

**Published:** 2023-07-10

**Authors:** Shaimaa A. Abdelrahman, Maha A. Khattab, Marian S. Youssef, Abeer A. Mahmoud

**Affiliations:** Medical Histology and Cell Biology Department, Faculty of Medicine, Zagazig University, Zagazig, Egypt

**Keywords:** Di-ethylhexyl phthalate, G-CSF, Cardiac muscle, Histology, Immunohistochemistry

## Abstract

Phthalates are common plasticizers present in medical-grade plastics and other everyday products. Di-ethylhexyl phthalate (DEHP) has been noted as a causative risk factor for the initiation and augmentation of cardiovascular functional disorders. G-CSF is a glycoprotein found in numerous tissues throughout the body and is currently applied in clinical practice and has been tested in congestive heart failure. We aimed to examine in depth the effect of DEHP on the histological and biochemical structure of the cardiac muscle in adult male albino rats and the mechanisms underlying the possible ameliorative effect of G-CSF. Forty-eight adult male albino rats were divided into control group, DEHP group, DEHP+ G-CSF group and DEHP-recovery group. We measured serum levels of aspartate aminotransferase (AST), creatine kinase MB isoenzyme (CK-MB) and lactate dehydrogenase (LDH). Left ventricular sections were processed for light and electron microscope examination, and immunohistochemical staining of Desmin, activated Caspase-3 and CD34. DEHP significantly increased enzyme levels, markedly distorted the normal architecture of cardiac muscle fibers, downregulated Desmin protein levels and enhanced fibrosis, and apoptosis. G-CSF treatment significantly decreased the enzyme levels compared to DEHP group. It enhanced CD34 positive stem cells recruitment to injured cardiac muscle, therefore improved the ultrastructural features of most cardiac muscle fibers via anti-fibrotic and anti-apoptotic effects in addition to increased Desmin protein expression levels. The recovery group showed partial improvement due to persistent DEHP effect. In conclusion, administration of G-CSF effectively corrected the histopathological, immunohistochemical and biochemical alterations in the cardiac muscle after DEHP administration by stem cells recruitment, Desmin protein regulation, antifibrotic and antiapoptotic mechanisms.

## Introduction

Di-ethylhexyl phthalate (DEHP) is a synthetic substance frequently used in consumer goods like soft squeeze toys, waxes, paints, solvents, building materials, medical equipment, electronics, personal care items, food products, and pharmaceuticals. Such products are anticipated to account for roughly 60% of global phthalate use by the year 2022 (Kim et al. 2018). In polyvinylchloride (PVC) formulations, DEHP; as one of phthalic acids, is a widely used phthalate plasticizer. Phthalic acids are oily liquids with moderate volatility. DEHP forms noncovalent connection with plastics, allowing for easy environmental release and quick anaerobic, photochemical, and biological breakdown (Cheon 2020).

Through eating, inhalation, and skin contact with phthalate-contaminated substances, phthalates are easily absorbed by humans. Dietary products with special packaging are thought to be the main source of phthalate exposure in the general population. The lipid content of the food, the packing procedure, the packing material, the time the food is in contact with the packing materials, and the storage temperature all affect how much DEHP is present in this type of food Wang et al. 2019). Different animal organ systems could be harmed by DEHP and its metabolites (Bansal et al. 2018). Several disorders such as testicular, ovarian, endocrine, renal, neurological, and hepatic toxicity are linked with them (Rowdhwal et al. 2018). Phthalates have been linked positively to the development of hypertension and atherosclerosis in adults and several cardiometabolic risk factors in children and adolescents. DEHP demonstrated negative effects on rat and chick embryonic cardiomyocyte function, resulting in electrophysiological alterations in the isolated rat heart and various effects on blood pressure (BP), including an increase in systolic and diastolic BP in 33-week-old rat progeny (Mariana, and Cairrao 2020).

Colony-stimulating factor 3 (CSF 3), also named Granulocyte Colony-Stimulating Factor (G-CSF), is a glycoprotein that is present in many body tissues. It could encourage cell survival, mobilization, and proliferation. It induces the production of many precursors, such as granulocytes and stem cells, in the bone marrow and releases them into the bloodstream. It helps ischemic cells survive in the central nervous system (CNS) and lowers the expression of pro-inflammatory cytokines (Aschauer-Wallner et al. 2021). G-CSF can improve heart function, stabilize the myocardial electrophysiological properties, prevent ventricular remodeling, minimize myocardial apoptosis and inflammation, and lessen the likelihood of ventricular arrhythmia following ischemia reperfusion injury (Wang et al. 2020; Hortu et al. 2019). It can stimulate neurogenesis in the CNS to promote neuroplasticity and prevent apoptosis. The neurotrophic effects of G-CSF were attributed by Abdel Mohsen et al. (2020) and Keiner et al. (2021) to its capacity to reduce oxidative stress secondary to accumulation of reactive oxygen species (ROS). Based on the aforementioned, the current study was carried out to investigate in detail how DEHP could affect the biochemical, histological, and immunohistochemical composition of the cardiac muscle in adult male albino rats and the various mechanisms underlying the potential ameliorative role of G-CSF.

## Materials and methods

### Animals

In this study, 48 adult male albino rats weighing 180–200 g were used. They were acquired from Zagazig Scientific and Medical Research Center (ZSMRC), Faculty of Medicine, Zagazig University. The Zagazig University Research Ethics Committee authorized the experimental protocol, which was carried out in compliance with the National Institute of Health's standards for using animals in research (IACUC approval number ZU-IACUC/3/F/28/2020). Before the experiment, the animals were kept for a week in stainless steel cages in the animal house to acclimate to the new environment. The rats were kept at room temperature with 12-h light/dark cycles for the duration of the experiment. They had unrestricted access to food and water. The Medical Histology & Cell Biology Department of Zagazig University's Faculty of Medicine carried out this investigation.

### Chemicals

DEHP was purchased from Sigma Chemical Company, St. Louis, MO, USA. Granulocyte colony-stimulating factor (Neupogen), A pre-filled syringe of 300 μg of filgrastim in 0.5 ml solution for injection (recombinant-methionyl human G-CSF, r-metHuG-CSF, from Escherichia coli K12) (F. Hoffmann-La Roche Ltd, Basel. Kirin-Amgen Inc., Switzerland). Desmin Polyclonal Antibody (Rabbit Polyclonal Antibody, Catalog No.: PA5-16705, Invitrogen, Thermo Fisher Scientific, Waltham, MA USA, 1:200 dilution). Caspase 3 (Cleaved Asp175) Antibody (Rabbit Polyclonal Antibody, Catalog No.: PA5-114687, Invitrogen, Thermo Fisher Scientific, Waltham, MA USA, 1:100 dilution). Anti-CD34 rat monoclonal antibody (ab8158, Abcam, Cambridge, MA, USA, 1:100 dilution).

### Experimental design

The rats were classified into four main groups. Group I (control group) included 18 rats that were equally subdivided into three subgroups, subgroup Ia (Negative control group) rats received no treatment till the end of the experiment. Subgroup Ib (Vehicle group), each rat received 1 ml of corn oil (solvent of DEHP) for 31 consecutive days by oral gavage, the same amount in which DEHP was dissolved. Subgroup Ic (Vehicle group) each rat received 0.5 ml of 5% glucose (solvent of G-CSF) by SC injection for 5 days. Group II (DEHP group) included 10 rats, they received DEHP dissolved in corn oil at a dose of 500 mg/kg/day by oral gavage for 31 consecutive days (Zhang et al. 2018) in the form of 1 ml corn oil /day for each rat containing 100 mg DEHP. Group III (DEHP+ G-CSF group) included 10 rats, they received DEHP as group II then by the end of DEHP exposure, rats were subcutaneously injected with G-CSF at a dose of 50 µg/kg/day (freshly dissolved in 0.5 ml of 5% glucose) for another 5 consecutive days (Omar et al. 2018). Group IV (DEHP-recovery group), ten rats received DEHP at the same dose and duration as group II. Then, they were left free without DEHP administration for another 31 days (the recovery period is equal to the duration of exposure to DEHP (Atia and Abdel-Gawad 2019).

According to each group's designated time, rats in each group were sacrificed. The intra peritoneal (IP) injection of sodium pentobarbital at a dose of 50 mg/kg body weight was used to anaesthetize rats. The heart was removed from the chest, rinsed with saline, and then bisected longitudinally after blood samples have been taken for biochemical examination. Samples from the left ventricle were prepared for histological and immunohistochemical investigations.

### Biochemical analysis

Blood samples were taken from the rats’ medial orbital vein into sterile syringes containing EDTA. All groups of rats had their serum tested for AST, CK-MB isoenzyme (CK-MB) activity, and lactate dehydrogenase (LDH) levels (Perez-Carceles et al. 1995).

### Light microscopic examination

Each animal's left ventricle was meticulously removed, and the specimens were then soaked in 10% formol saline for 48 h, dehydrated, and embedded in paraffin wax. To detect the morphological changes and collagen fibers deposition, paraffin sections were cut and stained with H&E and Mallory trichrome stain, respectively (Suvarna et al. 2018).

### Immunohistochemical study

To evaluate immunoexpression of Desmin protein, Activated Caspase-3 (as a marker for apoptosis) and CD34 (as a marker of stem cells) in the myocardium of albino rats** (**Iyer et al. 2014; Bei et al. 2015**).** Streptavidin–biotin complex immunoperoxidase system was used. Serial paraffin-embedded sections were deparaffinized on positively charged slides, incubated for 30 min in 0.1% hydrogen peroxide (to block the endogenous peroxidase) and then incubated with the corresponding primary antibody for 30 min at room temperature. Sections were then washed several times with PBS before being incubated with the secondary antibodies (biotinylated anti-rabbit or anti-rat IgG, Zymed Laboratories, South San Francisco, CA, USA). Staining was completed by incubation with chromogen, called Diamiobenzidine (DAB) (Ramos-Vara et al. 2008).

Positive reaction for Desmin appeared as a brown cytoplasmic coloration. Caspase-3 immunoexpression appeared as brown coloration that appeared either cytoplasmic or nuclear. Positive reaction for CD34 appeared as a brown cytoplasmic coloration in endothelial and interstitial cells. For negative control, the primary antibody was replaced by PBS. According to manufacturer’s instructions, the positive controls for the used antibodies were. Primary human hepatic stellate cells for Desmin Polyclonal Antibody (Cat# PA5-16705). Human Ehrlich carcinoma tissue for immunohistochemistry analysis of Caspase 3 (Cleaved Asp175). Anti-CD34 rat monoclonal antibody (ab8158) is selectively expressed on hematopoietic progenitor cells and the small vessel endothelium of a variety of tissues. Also lung tissue was used as a positive control.

### Transmission electron microscope (TEM) study

Sections from the left ventricle were cut into small pieces (0.5–1.0 mm^3^) for TEM ultrastructural analysis. Then, they were fixed in 2.5% phosphate-buffered glutaraldehyde (pH 7.4) followed by fixation in 1% osmium tetroxide in the same buffer at 4 °C, dehydrated, and embedded in epoxy resin. At the Electron Microscope Unit, Faculty of Agriculture, El Mansoura University, Egypt. Ultrathin sections were obtained using a Leica ultracut UCT, stained with uranyl acetate and lead citrate, examined, and photographed using a JEOL TEM 2100, Transmission Electron Microscope (Jeol Ltd, Tokyo, Japan) (Tizro et al. 2019).

### Image analysis and morphometric study

Sections stained for Desmin, Caspase-3 and CD34 were morphometrically analyzed. Data were obtained using Fiji image J (1.51n, NIH, USA) program at Medical Histology and Cell Biology Department, Faculty of Medicine, Zagazig University. The interactive measure menu was used to measure the area percent. The measuring frame was chosen so that the brown positive immune reaction could be seen and masked by blue binary color to be measured. Ten readings from five non-overlapping sections for each rat were examined.

### Statistical analysis

The statistical software for social sciences, version 21, was used to evaluate the recorded data (SPSS Inc., Chicago, Illinois, USA). The mean and standard deviation were used to express quantitative data (SD). Analysis of variance was used to test whether the experimental groups' mean values differed from one another (ANOVA). As a post hoc analysis of the ANOVA, Tukey's multiple comparison test was performed. When the P value was 0.05 or lower, the findings were regarded statistically significant. Different types of significance were considered.

## Results

### Effect of G-CSF on the DEHP-induced cardiac enzyme alterations

**Fig. 1:**
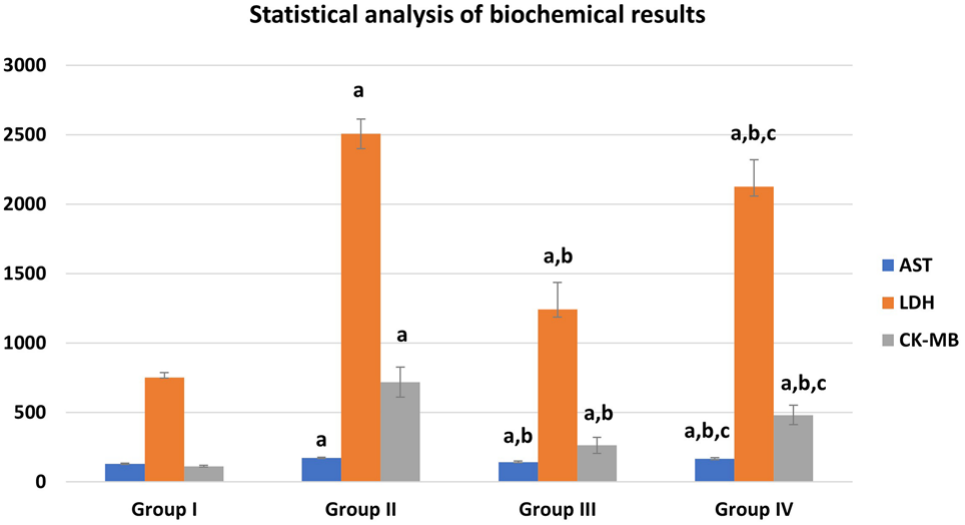
Biochemical results in different groups: **a** significant with control group I, **b** significant with DEHP group II, **c** significant with G-CSF group III

The mean values of AST, LDH, CK-MB were significantly increased in group II (DEHP-treated group) compared to other groups. They showed a statistically significant decrease in group III (G-CSF-treated group) compared to DEHP-treated group. The recovery group showed a significant increase in the three parameters compared to control and G-CSF-treated group III, and at the same times they showed significant decrease in their levels when compared to group II (Fig. 1).

### Effect of DEHP on the histological structure of the cardiac muscle

**Fig. 2:**
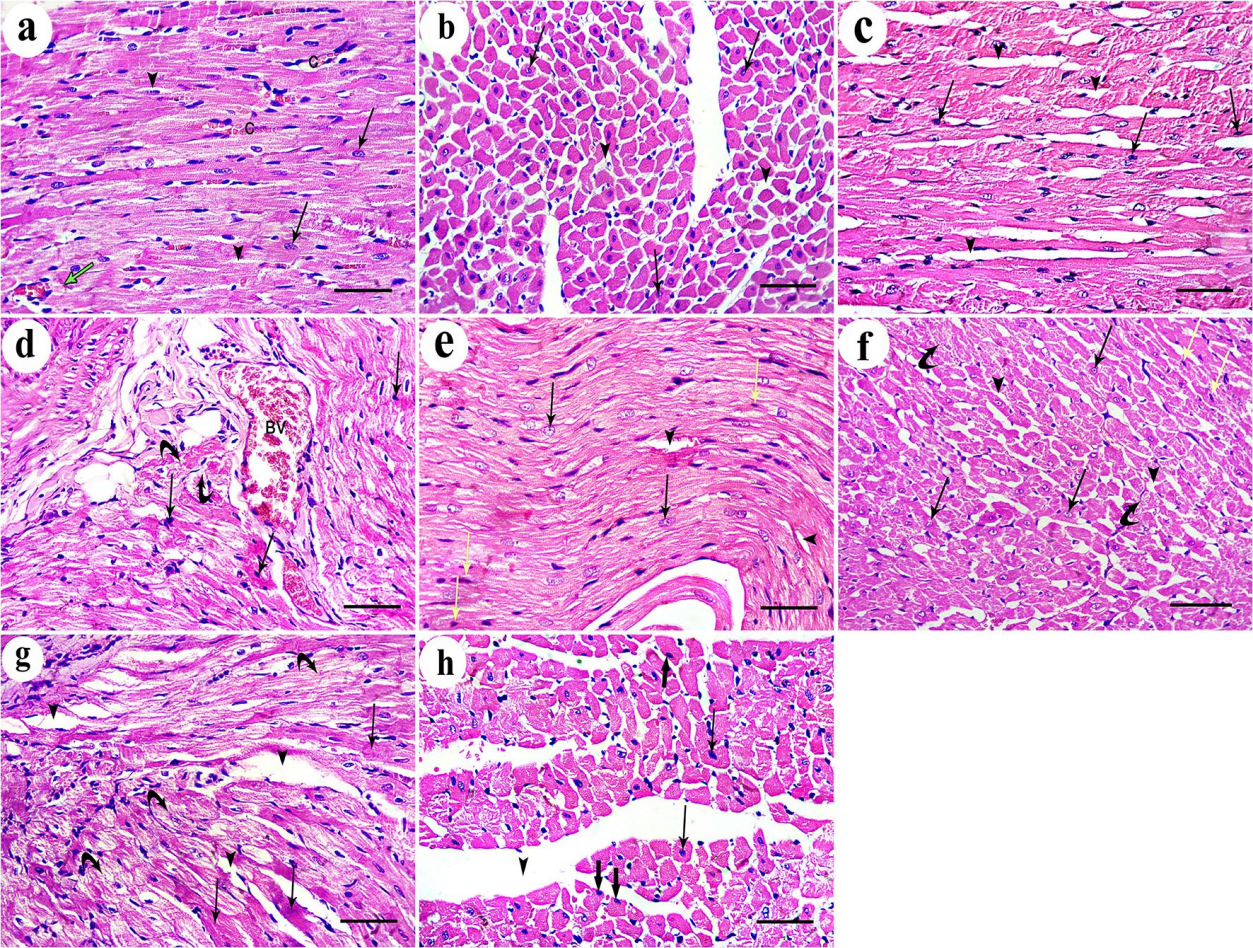
H & E-stained sections of rat cardiac muscle showing: **a** a longitudinal section of the control group shows branching cardiac muscle fibers with pale-stained central nuclei (arrows). Intercalated discs (green arrow) and narrow intercellular spaces (arrow heads) contain blood capillaries (C) are noticed. **b** A transverse section of the same group shows polygonal cardiac muscle fibers with central nucleus (arrows) and minimal intercellular spaces (arrow heads). **c** and **d** DEHP-treated group shows cardiac muscle fibers with darkly stained nuclei and cytoplasm (arrows) with disturbed myofilaments (curved arrows). Wide intercellular spaces (arrow heads) contain dilated and congested blood vessels (BV). **e** G-CSF-treated group shows most cardiac muscle fibers have pale stained oval nuclei (arrows) surrounded by acidophilic striated cytoplasm. A few of them show deeply stained nuclei and cytoplasm (yellow arrows). **f** A transverse section of the same group shows polygonal cardiac muscle fibers with central rounded nuclei and acidophilic cytoplasm (arrows). Few of them have dark nuclei (yellow arrows) and few myofilaments (curved arrows). Intercellular spaces (arrow heads) are narrow. **g** and **h** DEHP-recovery group shows deeply stained nuclei and cytoplasm (arrows), marked reduction in myofibrils (curved arrows) with complete loss of striations. Some cardiac muscle fibers have peripherally located nuclei (thick arrows) with wide intercellular spaces (arrow heads). (H&E × 400; Scale bar 30 µm)

**Fig. 3:**
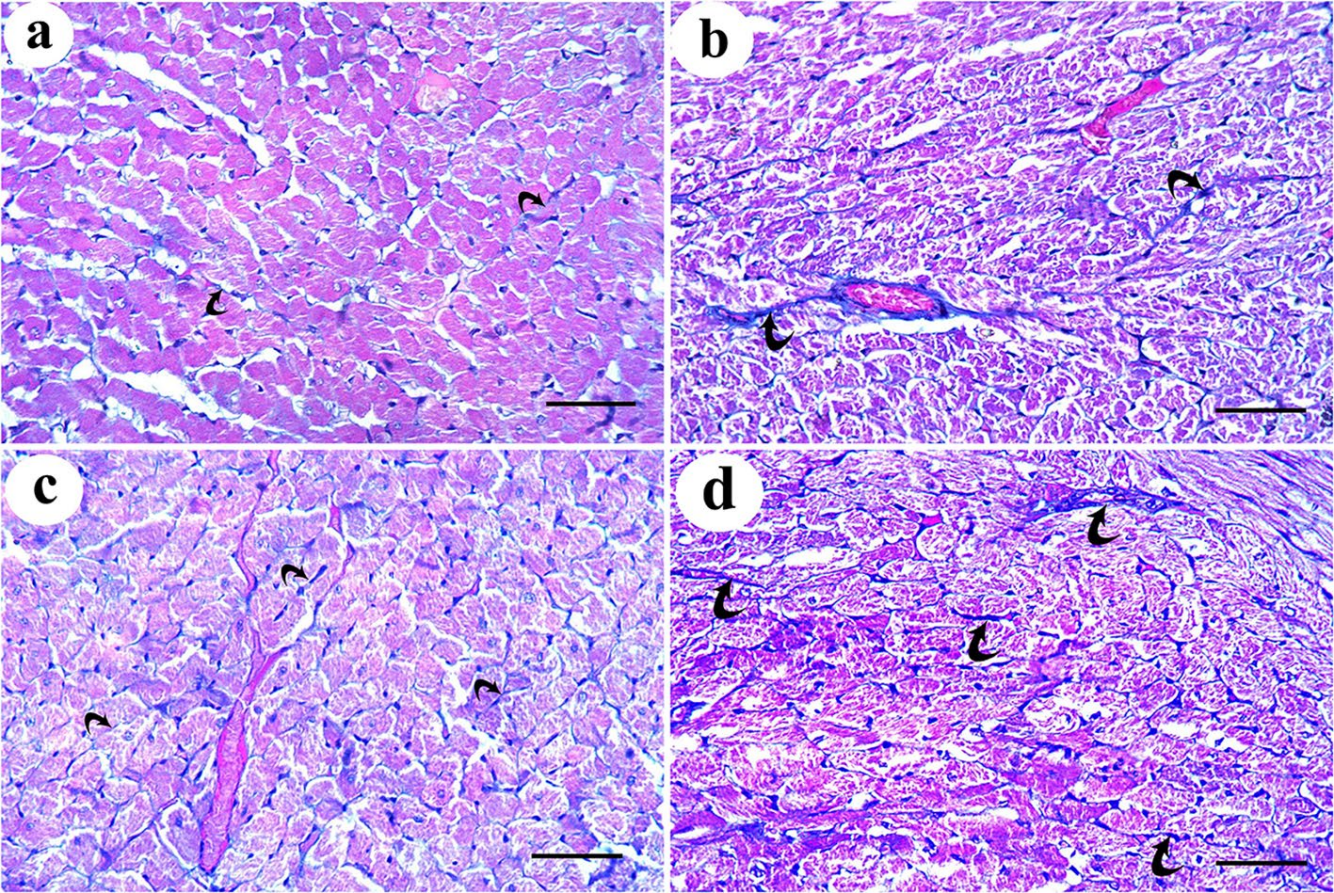
Mallory’s trichrome-stained sections show **a** delicate collagen fibers among cardiac muscle fibers of the control group. **b** Abundant collagen fibers aggregation in between the cardiac muscle fibers and around the congested blood vessels in DEHP-treated group. **c** Mild aggregation of collagen fibers in G-CSF-treated group. **d** Moderate aggregation of collagen fibers in DEHP-recovery group (Curved arrows indicate collagen fibers deposition). (Mallory’s trichrome stain × 400; Scale bar 30 µm)

Regarding the control group, the results of negative control group (Ia) were used to express group I results as no histologically significant differences were detected between the two subgroups. H&E-stained sections of control rat cardiac muscle showed the characteristic branching and interconnection pattern of neighboring cardiac muscle fibers. Each one had a pale stained oval centrally located nucleus surrounded by acidophilic striated cytoplasm. They were connected by intercalated discs and separated by narrow intercellular spaces containing small blood capillaries and in transverse section, they appeared rounded or polygonal with central nuclei (Fig. 2a, b). Mallory’s trichrome-stained sections showed delicate collagen fibers among the cardiac muscle fibers of the control group (Fig. 3a). DEHP-treated group showed marked disturbance in the normal architecture of cardiac muscle fibers. They were separated by wide intercellular spaces. Some of them appeared with small apoptotic nuclei and deeply stained cytoplasm with loss of striations. Others had marked reduction in myofilaments distribution. The blood vessels were dilated and congested (Fig. 2c, d). Abundant collagen fibers aggregate between the cardiac muscle fibers and around the congested blood vessels in DEHP-treated group (Fig. 3b).

### Effect of G-CSF on the DEHP-induced cardiac muscle injury

G-CSF-treated group showed preserved architecture of most cardiac muscle fibers. They had pale stained oval nuclei surrounded by acidophilic striated cytoplasm. Few of them showed deeply stained nuclei and cytoplasm. They appeared rounded or polygonal in transverse section with central rounded nuclei. Intercellular spaces were narrow (Fig. 2e, f). Mild aggregation of collagen fibers was observed between the cardiac muscle fibers and around the blood vessels in G-CSF-treated group (Fig. 3c).

### Effect of DEHP withdrawal on cardiac muscle structure

DEHP-recovery group showed some cardiac muscle fibers still had deeply stained nuclei and cytoplasm. Others had marked reduction in the aggregation of myofibrils with complete loss of their striations. Some cardiac muscle fibers showed peripherally located nuclei in transverse section. Intercellular spaces were wide (Fig. 2g, h). Moderate aggregation of collagen fibers was observed between the cardiac muscle fibers in DEHP-recovery group (Fig. 3d).

### Immunohistochemical and morphometric analysis

**Fig. 4:**
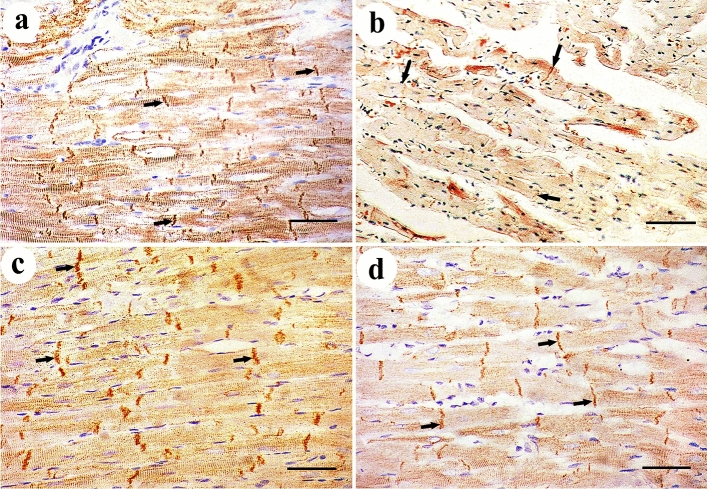
Desmin protein immunoexpression appears as dark brown color at the level of intercalated discs (thick arrows). **a** control group shows prominent Desmin expression. **b** DEHP group shows marked reduction in Desmin expression. **c** G-CSF-treated group shows prominent expression of Desmin while **d** DEHP-recovery group shows moderate Desmin expression. (Avidin biotin Peroxidase system × 400; Scale bar 30 µm)

**Fig. 5:**
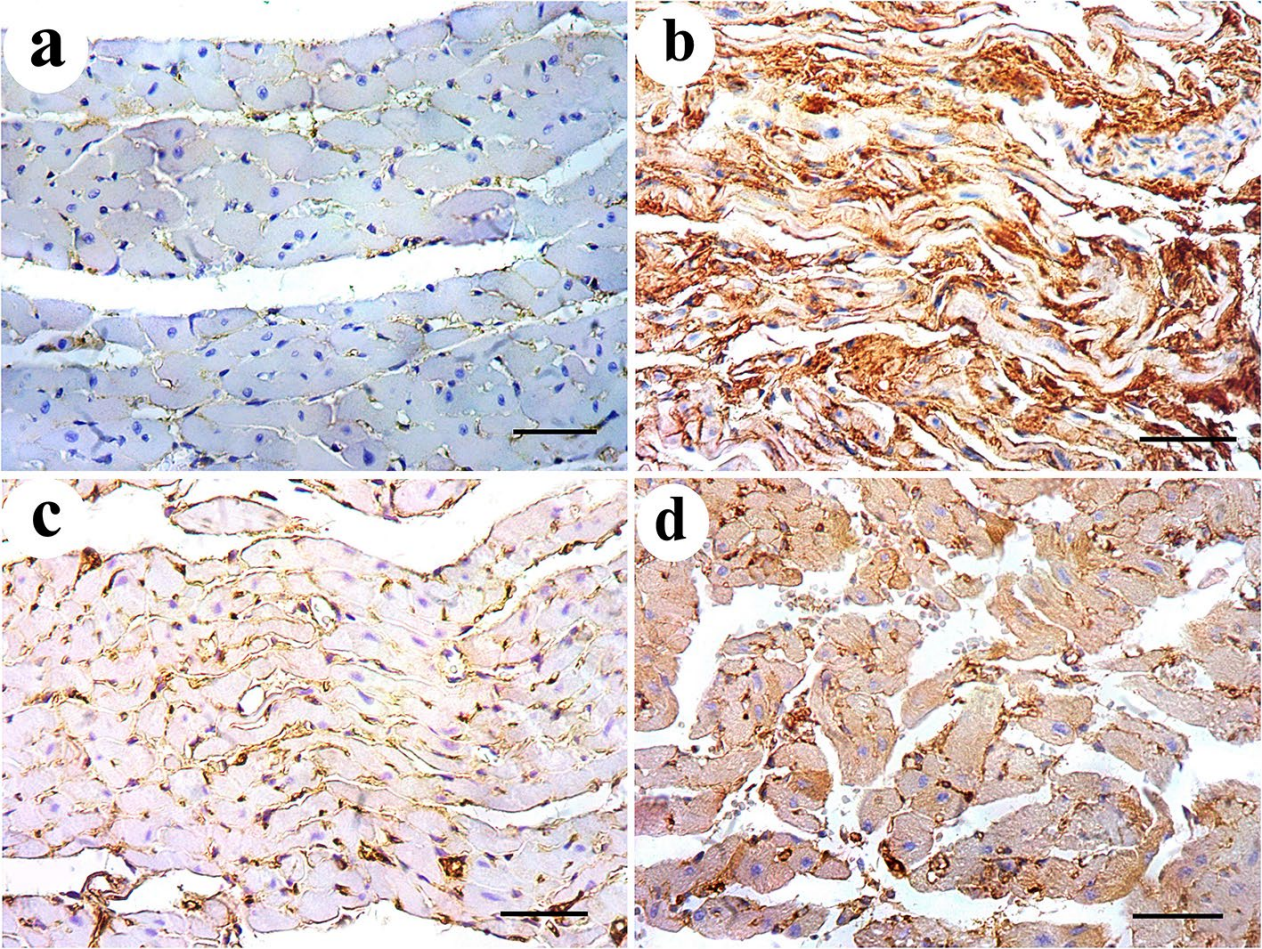
Caspase-3 immunoexpression shows **a** negative cytoplasmic expression for caspase-3 in the control group. **b** DEHP-treated group shows strong positive reaction in the cytoplasm of most cardiac muscle fibers. **c** G-CSF-treated group shows faint brown reaction in the cytoplasm of most cardiac muscle fibers. **d** DEHP-recovery group shows moderate caspase-3 expression in most cardiac muscle fibers. (Avidin biotin Peroxidase system × 400; Scale bar 30 µm)

**Fig. 6:**
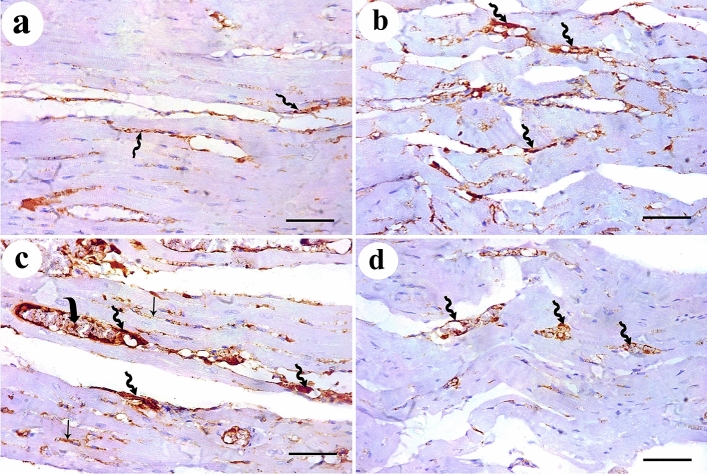
CD34+ ve stem cells immunoexpression shows **a** faint brown expression in the endothelial cells lining the blood vessels (zigzag arrow) in control group. **b** DEHP-treated group shows moderate immunoreaction in the endothelial cells lining the blood vessels (zigzag arrow). **c** G-CSF-treated group shows dark brown CD34 immunoreactions in the lumen (curved arrow) and endothelial cells lining the blood vessels (zigzag arrow) and in between the cardiac muscle fibers (arrow). **d** DEHP-recovery group shows moderate immunoreactions in the endothelial cells lining the blood vessels (zigzag arrow). (Avidin biotin Peroxidase system × 400; Scale bar 30 µm)

Immuno-histochemical expression of Desmin protein appeared as dark brown color at the level of intercalated discs in control group. DEHP-treated group showed marked reduction in Desmin expression. G-CSF-treated group showed prominent expression of Desmin while DEHP-recovery group showed moderate Desmin expression (Fig. 4). Immunohistochemical expression of caspase-3 showed negative expression of caspase -3 in the cytoplasm of all cardiac muscle fibers in control group. DEHP-treated group showed strong positive immunoreaction that appeared as a dark brown coloration in the cytoplasm of most cardiac muscle fibers. G-CSF-treated group showed faint brown immunoreaction in the cytoplasm of most cardiac muscle fibers. The recovery group showed moderate caspase-3 expression in most cardiac muscle fibers (Fig. 5). Immunohistochemical expression of CD34+ stem cells showed faint brown expression of CD34 immunoreactivity in the endothelial cells lining the blood vessels while cardiac muscle fibers showed negative reaction in control group. DEHP-treated group showed moderate brown immunoreactions in the endothelial cells lining the blood vessels. G-CSF-treated group showed dark brown CD34 immunoreactions in the lumen of the blood vessels, their endothelial cells lining, and in between the cardiac muscle fibers. The recovery group showed moderate immunoreactions in the endothelial cells lining the blood vessels (Fig. 6).

### Ultrastructural examination of the cardiac muscle

**Fig. 7:**
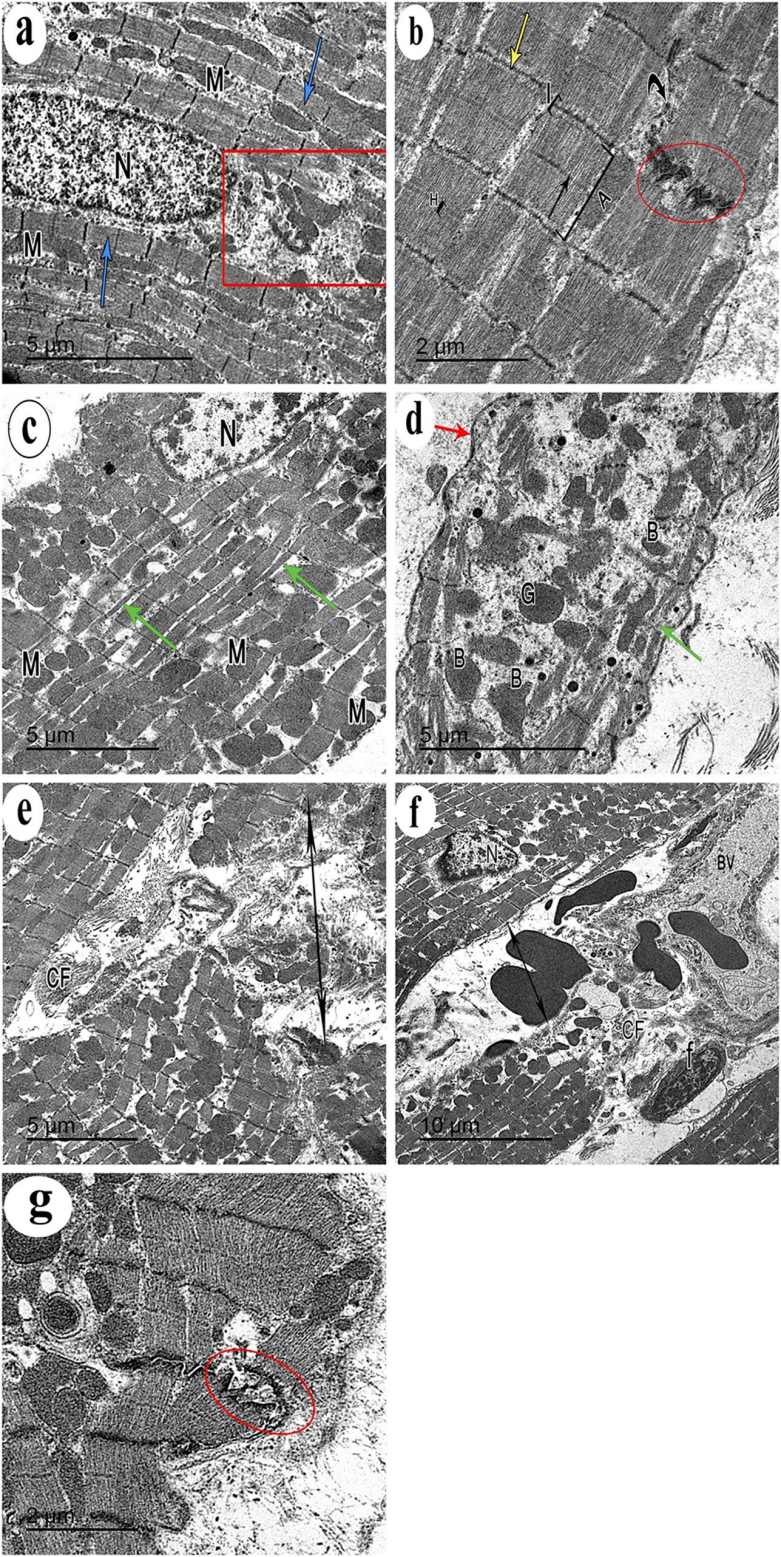
Transmission electron micrographs from rat cardiac muscles show **a** the control group shows a part of cardiac muscle cell with central oval euchromatic nucleus (N). Its sarcoplasm is packed with regular profiles of longitudinally arranged myofibrils (blue arrows) that are separated by thin layer of sarcoplasm containing rows of mitochondria (M). The myofibrils are diverted at the perinuclear area (red rectangle) leaving an obvious electron lucent area containing mitochondria. **b** The higher magnification of the same group shows the myofibrils are arranged in an alternative dark (A) and light (I) bands. The (A) bands have lighter (H) zones; that are bisected by dark (M) line (arrow). The (I) bands are traversed by dark Z-lines. An intercalated disc with both transverse electron dense portion (red circle) and longitudinal smooth segment (curved arrow) is also seen. **c** DEHP-treated group shows apart of cardiac muscle cell containing euchromatic nucleus (N) with patches of heterochromatin. Its sarcoplasm contains relatively attenuated myofibrils (green arrows). Among these myofibrils, numerous rows of variable sized mitochondria (M) are observed. **d** Another section of the same group shows cardiac muscle cell sarcoplasm containing widely separated Bizarre-shaped mitochondria (B) and empty electron lucent spaces in between them. Distorted attenuated myofibrils (green arrow) and corrugated sarcolemma (red arrow) are noticed. **e** DEHP-treated group also shows wide intercellular spaces (two headed arrow) among attenuated cardiac muscle cells containing prominent aggregations of collagen fibers (CF). **f** A wide intercellular space (two headed arrows) among cardiac muscle cells containing dilated congested blood vessels (BV) and **g** a widened distorted intercalated disc (red circle), can be observed in the same group. (TEM, Scale bars: a, c, d, e = 5 µm; b, g = 2 µm, and f = 10 µm)

**Fig. 8:**
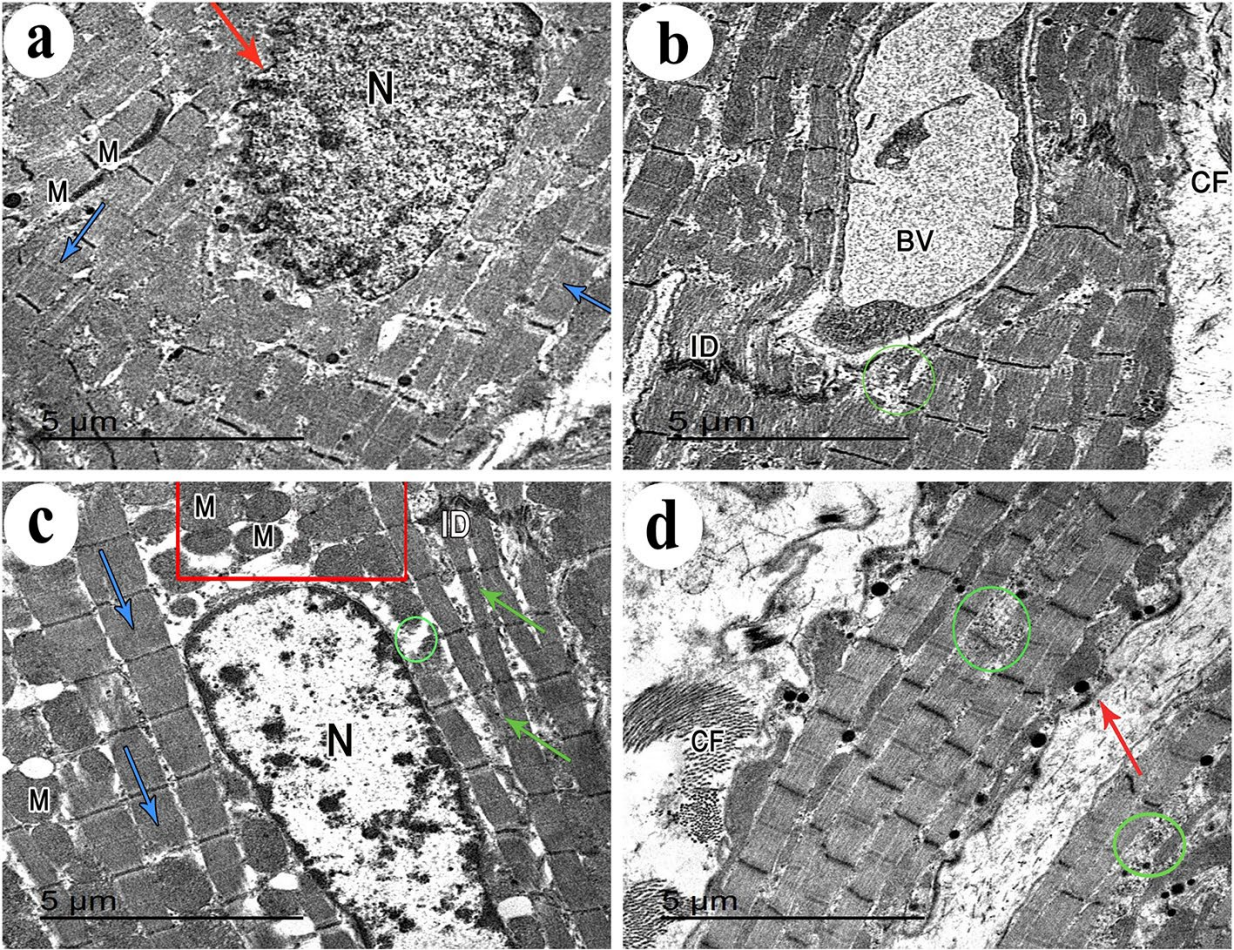
Transmission electron micrographs from rat cardiac muscles show **a** G-CSF-treated rats show part of cardiac muscle cell with ovoid euchromatic nucleus (N) with irregular nuclear envelope (red arrow). Its sarcoplasm is packed with well-formed myofibrils (blue arrows) which are separated by rows of flattened mitochondria (M). **b** Another section of the same group shows the intercellular space contains moderate aggregation of collagen fibers (CF) and blood capillaries (BV). Few areas of focal destruction (green circle) of myofibrils are noticed. Intercalated discs (ID) appear normal. **c** DEHP-recovery group shows a part of cardiac muscle fiber with ovoid euchromatic nucleus (N) showing peripheral thin rim of heterochromatin and surrounded by a perinuclear space (red rectangle) filled with aggregation of mitochondria (M). The sarcoplasm contains well-formed (blue arrows) and attenuated myofibrils (green arrows) with focal areas of myofilaments destruction (green circle). Intercalated disc (ID) is noticed. **d** Another section of the same group shows parts of cardiac muscle fibers surrounded by wide intercellular spaces containing prominent collagen fibers aggregation (CF). Focal destruction of myofibrils (green circles) and corrugated sarcolemma (red arrow) are noticed. (TEM, Scale bars = 5 µm)

Ultrastructural examination of the control group showed regular profiles of longitudinally arranged myofibrils separated by thin layer of sarcoplasm containing rows of mitochondria that were present also in the perinuclear area. The myofibrils were arranged in alternative dark (A) and light (I) bands. The (A) bands had lighter (H) zones; that were bisected by dark (M) line. The (I) bands were traversed by dark Z-lines. The intercalated disc showed both transverse electron dense portion and longitudinal smooth segment (Fig. 7a, b). Some mildly affected cardiac muscle fibers of DEHP-treated group showed euchromatic nuclei with patches of heterochromatin. The sarcoplasm had relatively attenuated myofibrils among which numerous rows of variable sized mitochondria were observed. Severely affected cardiac muscle fibers showed Bizarre-shaped mitochondria with empty electron lucent spaces in between them. The myofibrils were distorted and attenuated with corrugated sarcolemma. DEHP-treated group also showed wide intercellular spaces among attenuated cardiac muscle cells containing prominent aggregations of collagen fibers and dilated congested blood vessels. The intercalated discs were widened and distorted (Fig. 7c–g). G-CSF-treated rats showed cardiac muscle cells with ovoid euchromatic nuclei and irregular nuclear envelope. The sarcoplasm was packed with well-formed myofibrils which were separated by rows of flattened mitochondria. The intercellular space showed moderate aggregation of collagen fibers and blood capillaries. Few areas of focal destruction of myofibrils were noticed. Intercalated discs appeared normal (Fig. 8a, b). DEHP-recovery group showed cardiac muscle fiber with ovoid euchromatic nuclei showing peripheral thin rim of heterochromatin and surrounded by a perinuclear space filled with aggregation of mitochondria. The sarcoplasm showed both well-formed and attenuated myofibrils with focal areas of myofilaments destruction. The sarcolemma was corrugated. Intercalated discs appeared normal. Cardiac muscle fibers were surrounded by wide intercellular spaces containing prominent collagen fibers aggregation (Fig. 8c, d).

### Morphometric results

**Fig. 9:**
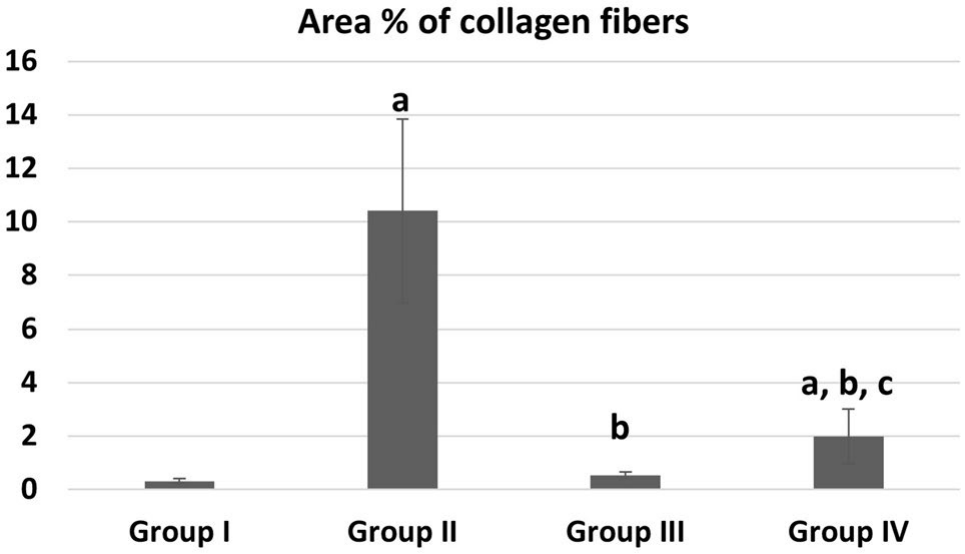
Area % of collagen fibers deposition in different groups. **a** Significant with control group I, **b** significant with DEHP group II, **c** significant with G-CSF group III

**Fig. 10:**
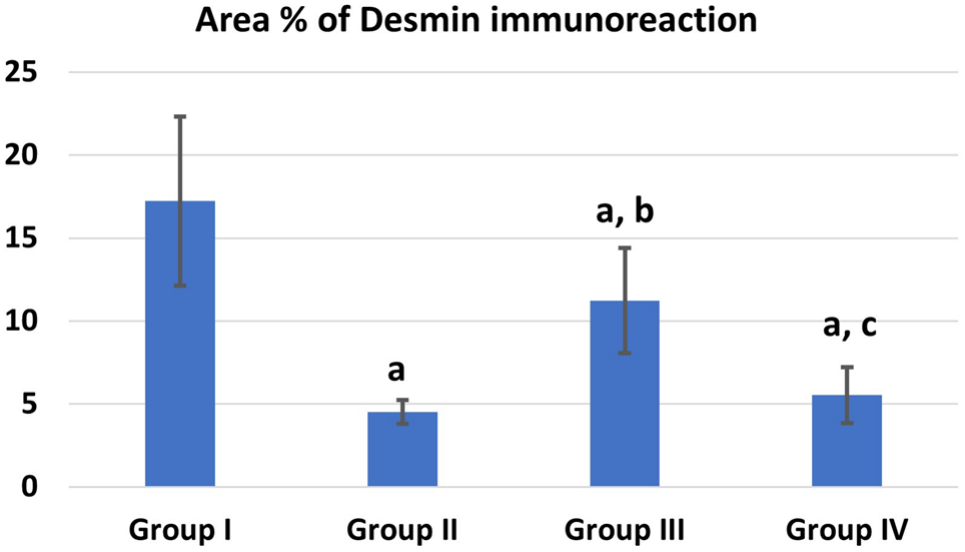
Area % of Desmin immunoreaction in different groups. **a** Significant with control group I, **b** significant with DEHP group II, **c** significant with G-CSF group III

**Fig. 11:**
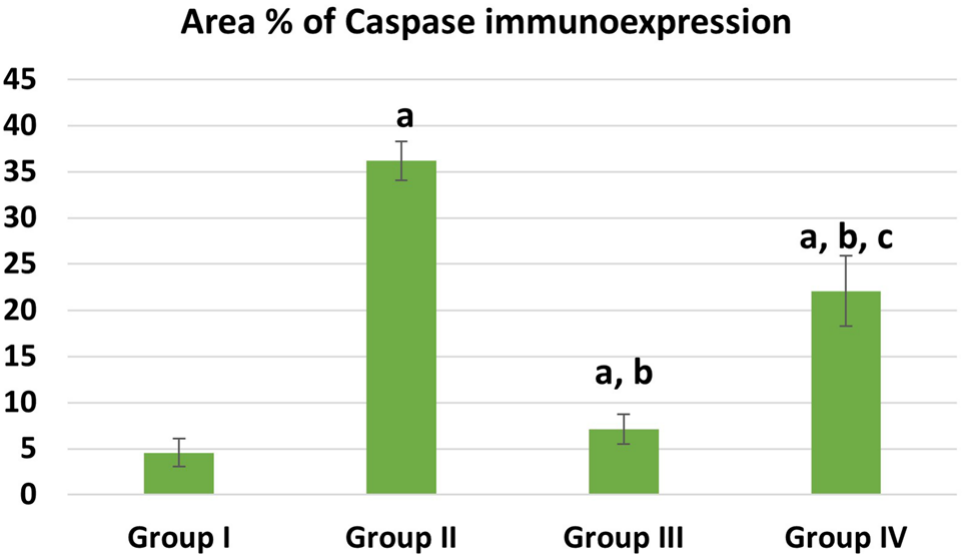
Area % of Caspase 3 immunoreaction in different groups. **a** Significant with control group I, **b** significant with DEHP group II, **c** significant with G-CSF group III

**Fig. 12:**
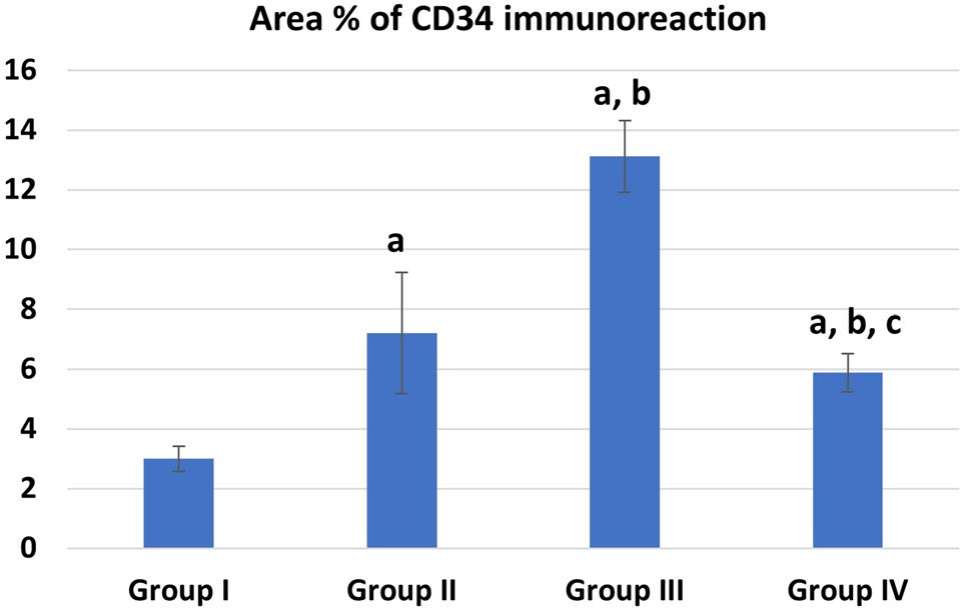
Area % of CD34 immunoreaction in different groups. **a** Significant with control group I, **b** significant with DEHP group II, **c** significant with G-CSF group III

The mean values of area % of collagen fibers deposition showed statistically significant increase (P ≤ 0.05) in groups II, IV than control. Groups III and IV showed a significant decrease (P ≤ 0.05) in collagen deposition than group II while recovery group IV showed more collagen deposition than group III (Fig. 9). The mean values of area % of Desmin immunoexpression showed statistically significant decrease (P ≤ 0.05) in groups II, III &IV than control. Group III showed a significantly increased (P ≤ 0.05) Desmin levels than group II. Recovery group IV showed nonsignificant (P > 0.05) difference from group II and at the same time it showed significant decreased Desmin levels than group III (P ≤ 0.05) (Fig. 10). The mean values of area % of Caspase 3 immunoexpression showed a statistically significant (P ≤ 0.05) increase in groups II, III &IV than control. Groups III and IV showed decreased expression levels than group II (with marked improvement observed in group III). Recovery group IV showed less improvement in Caspase expression (highly significant with group III, (P ≤ 0.001) (Fig. 11). CD34 immunoexpression levels showed statistically significant increase (P ≤ 0.05) in groups II, III &IV than control with marked increase in their levels observed in group III (P ≤ 0.001). Recovery group IV showed a significant decrease in CD 34 expression than groups II, III (P ≤ 0.05) (Fig. 12).

## Discussion

In the present study, corn oil was used as a vehicle to dissolve DEHP due to the lipophilic character of phthalates and the efficient absorption of corn oil from gastrointestinal mucosa which assures appropriate absorption of DEHP dosage (Takai et al. 2009; Engel and Wolff 2013; Farag et al. 2021) DEHP group revealed marked disturbance in the normal architecture of cardiac muscle fibers. Kasahara et al. (2002) reported that DEHP exposure can induce oxidative stress of cardiac muscle cells resulting in imbalance between reactive oxygen species (ROS) production and cellular antioxidants as glutathione and ascorbic acid. Mariana and Cairrao 2020 added that, ROS inhibitors can protect cardiac muscle cells against DEHP. From another point of view, Miura et al. (2007) attributed these changes to the over production of nitric oxide (NO), which causes damage of cellular lipids, proteins, and DNA and subsequently cell death.

Ultrastructural examination of the cardiac muscle fibers revealed heterochromatic nuclei and areas of focal destruction and even loss of myofilaments in DEHP group. Severely affected sections showed Bizarre-shaped mitochondria with empty spaces in between them. Our findings agreed with that of Zhang et al. (2022) and Miranda et al. (2003) DEHP harms mitochondria and inhibits nuclear respiratory factor 1 (Nrf1)-mediated mitochondrial biogenesis, resulting in mitochondrial damage. Bell and Hubert (1980) previously reported DEHP accumulation in the cardiac mitochondria with inhibition of adenine nucleotide translocase. It is one of the mitochondrial translocators located in the inner mitochondrial membrane and is responsible for molecular exchange of extra mitochondrial ADP to intra mitochondrial ATP. Increased permeability of mitochondrial membrane also leads to release of cytochrome C into the cytosol and activation of procaspases to caspase 3 and 7 which are hallmarks of apoptosis (Ouyang et al. 2012). Such effects of DEHP may be the underlying mechanism of myocardial cell death. Yu et al. (2022) attributed these changes to excess hydrogen peroxidase (H_2_O_2_) and ROS.

DEHP results also in membrane lipids peroxidation leading to breakdown of the sarcolemma membrane, promoting inflammation and necrosis (Howard et al. 2011; Kitmitto et al. 2019). Demonbreun et al. (2019) stated that, instability of myocyte plasma membrane contributes to dysregulation in calcium homoeostasis. In addition, the intercalated discs were widened and distorted in our study, as DEHP adversely affected the synchronization of cardiac cell network by disrupting connexin-43, the main component of cardiac gap junctions (Gillum et al. 2009). This effect suggests an arrhythmogenic effect of phthalates in vitro due to modifications in tubulin and kinesin, as well as other gene expression modifications (Posnack et al. 2011).

Administration of G-CSF following DEHP exposure in the present work markedly ameliorated the myocardium structure and enzyme levels. G-CSF was administered to rats by SC route of administration as it does not increase patients’ suffering or decrease their quality of life in hospitalized patients receiving chemotherapy according to (Paul et al. 2014). The duration of G-CSF administration in our study was only 5 days according to Omar et al. (2018) they stated that maximum mobilization of BM-HSCs occurs after 4–6 days of G-CSF administration. This clarifies that, the effect of G-CSF and the mobilized stem cells in cardiac tissue is paracrine due to the short period of administration which is not sufficient for the mobilized MSCs to be differentiated into cardiomyocytes.

Our findings agreed also with Baldo et al. (2011), who reported that G-CSF has beneficial effects on myocardial regeneration, in the form of acceleration of wound healing and the suppression of myocardial apoptosis. It is also responsible for the return of cardiac function after a myocardial infarction (Theiss et al. 2013) and recovery in ischemia illness models (Deindl et al. 2006).In addition to the fact that G-CSF mobilizes stem/progenitor cells to the periphery, these cells can move to the inflamed heart and contribute to tissue regeneration. The mobilized stem cells under the effect of G-CSF, were detected in the present study by immunohistochemical examination of CD34-positive stem cells which showed increased expression both in the lumen and in the lining endothelial cells of the blood vessels. In addition, increased immune expression of these cells was noticed in between the cardiac muscle fibers of the same group III. The selective recruitment of MSCs to injured tissue occurs by trans-endothelial migration directed by chemokine gradient (Fox et al. 2007). According to Fukuhara et al. (2004), the mobilized BM-MSCs could repair the injured myocardium. Since these cells are putative stem or progenitor cells therefore, they could improve myocardial perfusion, neovascularization, and regeneration when brought in contact with damaged myocardium according to Ripa (2011).

When rats were left without DEHP exposure in the recovery group, they displayed partial improvement in the histological structure of ventricular cardiomyocytes. This could be attributed to partial disruption of connexin-43; the primary component of cardiac gap junctions, during a period of DEHP exposure and hence impairs the synchronization of a cardiac cell network (Gillum et al. 2009). After stopping DEHP administration for 2 weeks in the study of Atia and Abdel-Gawad (2019), they reported mild improvement in the lung tissue, but complete recovery was not achieved. David et al. (2001) have also suggested that DEHP acts on organs via an adaptive mechanism that is reversible. The reversibility of their findings coincided with metabolic changes associated with the decrease in peroxisomal enzyme activity.

DEHP-exposed group revealed significant collagen fibers deposition both in-between cardiac muscle fibers and around the congested blood vessels. Ishizu et al. (2014) stated that the prominent collagen fibers in myocardium are type I and type III. Collagen fibers type I provide cardiac rigidity while type III provides more elasticity. Nishikawa et al. (2001) found that DEHP induces abundant collagen type I and elevates collagen I/III ratio with subsequent increased rigidity of the heart wall. Baum and Duffy (2011) reported that pathological stimuli can enhance secretion of collagen fibers and TGF-β from resident cardiac fibroblasts. Amara et al. (2019) and Liu et al. (2019) attributed fibrosis in DEHP group to the occurrence of oxidative stress through production of ROS. On the other hand, the significant decrease in fibrosis after G-CSF administration was explained by Sugano et al. (2005) who attributed it to an increase in reparative collagen production in damaged areas. After myocardial infarction, a decrease in fibrosis was also reported following long-term treatment with moderate doses of G-CSF in the study of (Okada et al. 2008). G-CSF induces MMP-2 (gelatinase A/type IV collagenase) and MMP-9 (gelatinase B) (Elsässer 2000). Cessation of DEHP in the recovery group showed moderate aggregation of collagen fibers. It might be attributed to a reduction of type I collagen protein in the left ventricular sections as well as a decrease in collagen I/III ratio, with improved flexibility and decreased fibrosis according to (David et al. 2001).

In the present study, marked reduction in the cytoplasmic expression of Desmin protein was noticed in DEHP group compared to control. Wang et al. (2002) and Dalakasa et al. (2003) reported that Desmin (a muscle specific intermediate filament protein expressed in cardiac, skeletal, and smooth muscles), is a key subunit of the intermediate filament in cardiac muscles. It interacts with other cytoskeleton proteins such as vimentin, nestin and lamin forming a 3-dimensional cytoskeletal network. McLendon and Robbins (2011) added that Desmin plays a crucial role in maintaining the structural and mechanical integrity of the contractile apparatus in muscle tissues. It links the contractile apparatus to the rest of the myocyte (the sarcolemma, extracellular matrix, and the nuclear lamina). It also maintains structural interactions at the Z-discs and intercalated discs. Moreover, Desmin filaments interact with the mitochondria, ensuring their proximity to the A and I band in the sarcomere. Thus, Desmin maintains the architectural organization of the myofibrils, influences organelle positioning, mediates organelle trafficking, cell to cell communication and signaling. Therefore, both overexpression and absence of Desmin have been linked to cardiac diseases.

The mechanism underlying the effect of DEHP on Desmin levels was explained by Tang et al. (2018) and Sun et al. (2021). They stated that DEHP can down regulate GATA4 expression, which is a key transcriptional factor in cardiac development and controls the expression of Desmin protein in the heart. Pawlak et al. (2009) reported that low levels of cardiac Desmin in heart failure patients were associated with bad prognosis. Also, Pawlak et al. (2012) proved that mice lacking Desmin usually exhibit architectural and ultrastructural abnormalities in the heart. Therefore, we can deduce that, the different ultrastructural changes occurred in the cardiac myocytes of DEHP group of our study were in part related to alterations in the expression levels of Desmin protein as proved by immunohistochemistry.

In comparison to the DEHP group, G-CSF significantly restored the expression of Desmin. According to Li et al. (2006) and Li et al. (2007) G-CSF treatment promotes the activation of Stat3 and Akt, which leads to the overexpression of GATA-4. This provides an explanation of the molecular mechanisms underlying the positive impacts of G-CSF on the failing post-MI heart. Moreover, the recovery group (IV) showed minimal amelioration in Desmin levels relative to group (II) with significant difference from groups I and III. Once the recovery group stopped being exposed to DEHP, cardiac GATA4 expression started to go back to normal (Atia and Abdel-Gawad 2019). The interaction between the intermediate filaments (IFs) in muscle cells is responsible for maintaining cell structure, organelle positioning, cell movement and differentiation. In addition, it impacts cellular signaling and gene expression in response to stressful stimuli (Etienne-Manneville and Lammerding. 2017; Tsikitis et al. 2018). According to Ralston et al. (2006) and Mattioli et al. (2011) Desmin and type A Lamins contribute to nuclear positioning, which specifies myonuclear domains. Consequently, altered myonuclear positioning characterizes both Desmin null muscle fibers and laminopathic muscles. These findings could also explain the presence of peripherally located nuclei in some H&E-stained sections of recovery group of the present study.

In the present study, DEHP induced cardiac apoptotic effect that was confirmed by strong positive expression of activated Caspase 3. DEHP and its metabolites MEHP were previously proved to activate Caspase 3 followed by induction of apoptotic cell death (Farag et al. 2021; Aung et al. 2014; Behairy et al. 2021). Recent studies explained the mechanism of DEHP-induced apoptosis and cell cycle arrest in a model of CVD, by increasing expressions of peroxisome proliferator-activated receptor (PPAR) and thereby inhibiting the phosphatidylinositol 3-kinase (PI3K)/threonine-protein kinase (AKT) survival pathway in differentiated human embryonic stem cells (Parrotta et al. 2020; Wen et al.^2022^). On the other hand, G-CSF markedly reversed DEHP–induced apoptosis. The effects of G-CSF therapy may be partially attributable to the modulation of abnormal immune responses. In addition, these findings could be owed to anti-inflammatory and anti-apoptotic properties of G-CSF (Pastuszko et al. 2017). G-CSF suppresses T cells by inducing apoptosis as mentioned by Peng (2017). Macambira et al. (2009) showed that G-CSF therapy ameliorated myocardial fibrosis and inflammation, and improved electrocardiography (ECG) abnormalities in a mouse model of Chagas disease cardiomyopathy.

Functional amelioration with G-CSF was also proved biochemically by significant decrease in AST, LDH and CK-MB activities which showed nearly normal serum levels of cardiac enzymes. These findings could be owed to the effect of G-CSF on promoting the proliferation of developing cardiomyocytes, as well as protecting the cellular membrane probity from oxidative damage and restoring the antioxidant system which coincide with the results of Lim et al. (2011). The recovery group also showed a significant decrease in cardiac enzymes compared to DEHP-treated group. Meanwhile, serum levels of these enzymes were still significantly different from groups I and III which indicates partial improvement in cardiac muscle. This might be attributed to the re-generation of ventricular muscle fibers and cardiomyocyte repair upon stopping exposure to DEHP and the subsequent stopping of the release of its enzymes into the blood (David et al. 1999). Mu et al. (2020) and Pu et al. (2020) reported that improvement after cessation of DEHP exposure depends on several factors such as the dose of DEHP, the duration and time of exposure either pre- or post-natal and finally depends on the animal species.

In conclusion, our results endorse our assumption that G-CSF has a propitious protective effect in correcting the DEHP-induced cardiac alterations, both by its effect on cardiomyocytes and by recruiting stem cells from bone marrow. We proved the ameliorative effect of G-CSF was through regulation of cardiac Desmin protein levels in addition to inhibitory effect on cardiac muscle fibrosis and apoptosis. With no recorded adverse effects of G-CSF at the levels utilized in this trial, our findings indicate that treatment with G-CSF may be regarded as a potentially novel option for the treatment of DEHP-induced cardiotoxicity.

## Data Availability

All data generated or analyzed during this study are included in this article. Further enquiries can be directed to the corresponding author.
